# Cell blebbing novel therapeutic possibilities to counter metastasis

**DOI:** 10.1007/s10585-024-10308-z

**Published:** 2024-09-02

**Authors:** Weiyi Jia, Marcus Czabanka, Thomas Broggini

**Affiliations:** 1 Department of Neurosurgery, University Hospital Frankfurt, Goethe University Frankfurt, Frankfurt, Germany; 2 Frankfurt Cancer Institute (FCI), Goethe University Frankfurt, Frankfurt, Germany

**Keywords:** Blebbing, Metastasis, Cell migration, Cytoskeletal dynamics, Cancer therapy, Myosin II migration

## Abstract

Cells constantly reshape there plasma membrane and cytoskeleton during physiological and pathological processes (Hagmann et al. in J Cell Biochem 73:488–499, 1999). Cell blebbing, the formation of bulges or protrusions on the cell membrane, is related to mechanical stress, changes in intracellular pressure, chemical signals, or genetic anomalies. These membrane bulges interfere with the force balance of actin filaments, microtubules, and intermediate filaments, the basic components of the cytoskeleton (Charras in J Microsc 231:466–478, 2008). In the past, these blebs with circular structures were considered apoptotic markers (Blaser et al. in Dev Cell 11:613–627, 2006). Cell blebbing activates phagocytes and promotes the rapid removal of intrinsic compartments. However, recent studies have revealed that blebbing is associated with dynamic cell reorganization and alters the movement of cells in-vivo and in-vitro (Charras and Paluch in Nat Rev Mol Cell Biol 9:730–736, 2008). During tumor progression, blebbing promotes invasion of cancer cells into blood, and lymphatic vessels, facilitating tumor progression and metastasis (Weems et al. in Nature 615:517–525, 2023). Blebbing is a dominant feature of tumor cells generally absent in normal cells. Restricting tumor blebbing reduces anoikis resistance (survival in suspension) (Weems et al. in Nature 615:517–525, 2023). Hence, therapeutic intervention with targeting blebbing could be highly selective for proliferating pro-metastatic tumor cells, providing a novel therapeutic pathway for tumor metastasis with minimal side effects. Here, we review the association between cell blebbing and tumor cells, to uncover new research directions and strategies for metastatic cancer therapy. Finaly, we aim to identify the druggable targets of metastatic cancer in relation to cell blebbing.

## Introduction

**Fig. 1:**
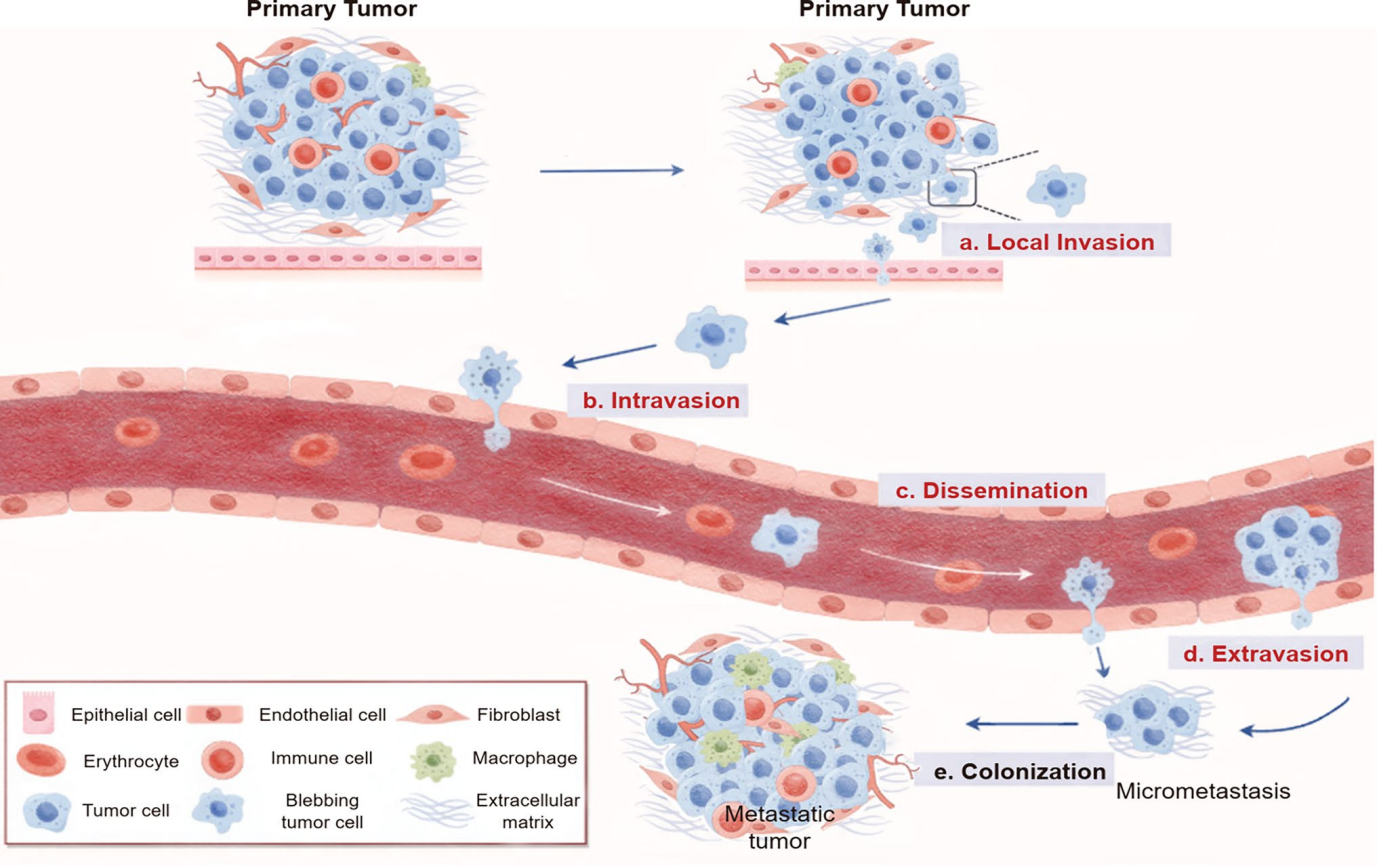
The blebbing phenomenon in tumor cells is evident throughout the entire process of tumor metastasis, encompassing various stages. **a** Local Invasion: Cells located at the primary tumor site exhibit blebbing as they invade the surrounding stroma; **b** Intravasation: Cancer cells undergo blebbing as they intrava-sate, entering the bloodstream; **c** Dissemination: Blebbing is observed as cancer cells travel through blood vessels to reach distant sites within the body; **d** Extravasation: Upon reaching a secondary tissue, cancer cells exhibit blebbing during the process of extravasation from the blood vessel; **e** Colonization: Finally, in the secondary tissue, blebbing is part of the cellular behavior involved in the formation of metastasis, marking the colonization phase

Cellular blebbing was first described as hyaline blisters or blebs [1–6], and later as smooth, rounded protrusions of the cytoplasmic membrane [2]. Blebs are well described feature of apoptosis [7]. However, recently blebs were discovered in different cell types unassociated with physiological and pathological conditions, hey are increasingly related to cell migration [4, 8–10]. This novel mechanism of cellular migration involves the formation of blebs, also known as amoeboid motility, which is typically associated with the suppression of lamellipodia extension and contraction [11, 12]. The mechanism has been observed in various contexts, such as in zebrafish primordial germ cells [3, 13]. Lamellipodia are actin filaments influencing cell polarization by shaping leading migratory edge of mesenchymal cells. Cells transition between different kinds of protrusions to migrate through gaps in lymphatic and blood vessels using lamellipodia [14] an indispensable feature found in metastatic cancer cells. In the context of tumor metastasis, cancer cells undergo a multi-step process that includes local invasion, intravasation into the bloodstream, survival in the circulatory system, extravasation into distant tissues, and colonization to form secondary tumors [15, 16] (Fig. 1). The migratory abilities of cancer cells using are crucial for their invasive and metastatic capabilities [17] (red titles in Fig. 1).

**Fig. 2:**
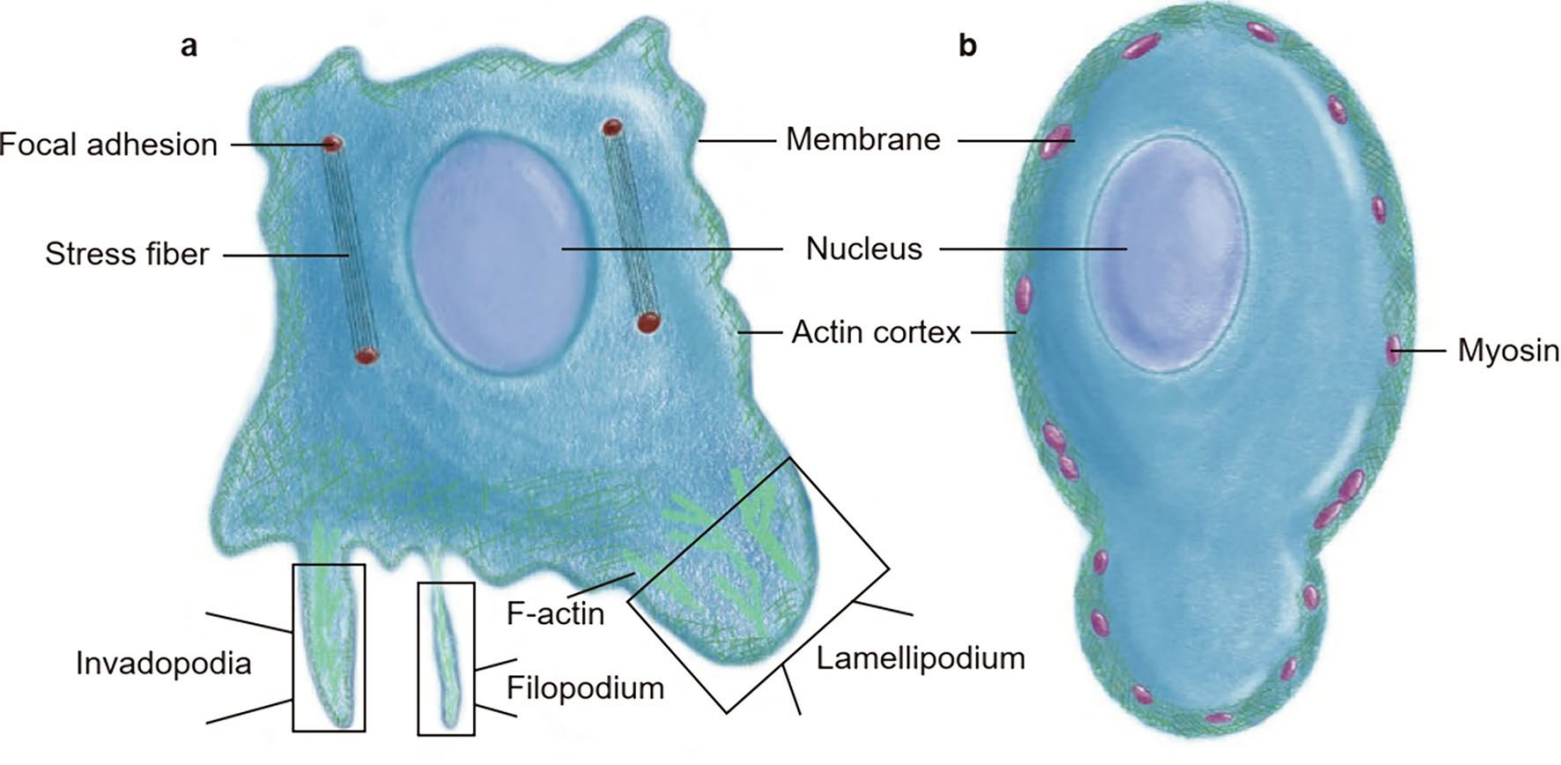
Mesenchymal migration and bleb-based amoeboid migration represent distinct modes of cell movement. **a** Cells engaged in mesenchymal migration employ actin-enriched protrusions, such as lamellipodia, particularly on rigid substrates that facilitate robust adhesion; **b** Conversely, when cells inhabit pliant environments like collagen gels or confined spaces, they opt for blebs to execute amoeboid migration. A bleb is a cellular membrane structure lacking the actin cortex. Following bleb genesis, it expands in response to cytoplasmic influx until the actin cytoskeleton reassembles beneath the plasma membrane (PM), halting further expansion. Subsequently, the bleb undergoes retraction propelled by the contractile force generated by actomyosin

**Fig. 3:**
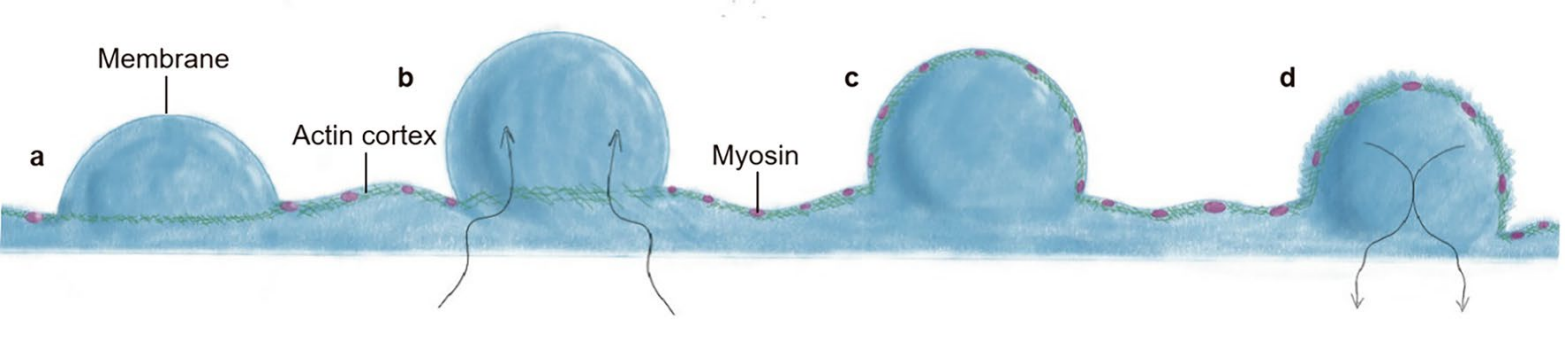
The bleb life cycle involves three primary stages: initiation, expansion, and retraction. **a** In the initiation stage, local disruption of the cortex–membrane interaction leads to the rapid formation of a large-volume plasma membrane (PM) protrusion promoted by cytoplasmic hydrostatic pressure, resulting in the rapid formation of blebs; **b** Hydrostatic pressure in the cytoplasm propels membrane expansion, either through the remaining cortex or through cortical pores; **c** The membrane further detaches from the cortex, increasing the diameter of the bleb base. As bleb expansion slows down, a new actin cortex forms under the bleb membrane; **d** The recruitment of myosin to the new cortex is followed by bleb retraction. Increased assembly of actin filaments, recruitment of myosin to the bleb lumen, and other factors generate contractile forces, leading to bleb retraction

Directional, mesenchymal migration is achieved by lamellipodia formation and movement towards the cellular translocation site [18] (Fig. 2a). In contrast, blebs are spherical membrane projections. During migration, these membrane structures display a high degree of deformability and produce a variety of protrusions [19]. Unlike lamellipodia, blebs are initially F-actin negative but later become F-actin positive, altering their stability and function. Furthermore, they rely on non-muscle myosin II (NMII)-mediated contractility to produce hydrostatic pressure [4] (Fig. 2b). Cellular blebs form and disappear in three phases: initiation, growth, and contraction. Blebs initiate when an extracellular stimulus triggers the plasma membrane to start separating from the actin cortex below (Fig. 3a, b). Subsequently, actinomyosin contractility generates hydrostatic pressure in the cytoplasm. The volume and surface area of the blebs expand after cytoplasmic and lipids influx through the narrow neck [4] (Fig. 3c). As the rate of actinomyosin contractility-dependent fluid and lipid influx becomes insufficient to maintain sustained growth, bleb enlargement gradually subsides [20] (Fig. 3d).

The advancements of blebs for cell motility render them interesting for metastatic research [21–23]. Recent studies have demonstrated that blebs play a facilitating role in metastatic cancer [24]. Although some research has confirmed the importance of these protrusion for cancer cell invasion, the fundamental mechanisms still need to be investigated [25]. The evidence that blebs have a specific and critical function in tumor cell metastasis and invasion is incomplete. This review discusses the mechanisms for bleb formation, the role of these protrusions in cytogenetic motility, and the relationship between bleb formation and restriction in the presence of various stimuli and different therapeutic agents.

## The mechanics of bleb formation

The initiation, expansion and stability maintenance of blebs are key processes affecting cell migration. Blebbing activity is divided into three stages: initiation, expansion, and retraction [10]. Bleb initiation is caused by the plasma membrane separating from the underlying actin cortex. Afterwards, during the growth phase, the flow of actin-free cytoplasm causes a larger membrane region to become further detached from the actin cortex [26]. Additional actin cortex eventually forms at the plasma membrane of the bleb, slowing the expansion. The contraction of this newly formed cortex may then cause the bleb to retract [20]. Hence, bleb formation is dependent on biophysical properties of the cell and correlates with intracellular hydrostatic pressure (Fig. 3).

### Bleb initiation

Two mechanisms are involved in bleb initiation: a localized reduction of membrane material followed by a reduction of actin cortex [26], or a localized rupture of the actin cortex itself [27]. Cell blebbing can be triggered artificially by mechanical stimulation (rapid aspiration through micropipettes) [28, 29], optical stimulation (laser ablation), as well as pharmacologically (actin-depolymerizing drugs) [30]. All stimuli cause a local reduction or rupture of the actin cortex membrane bond. However, the physiological mechanisms underlying the initiation of blebbing in migration are still unknown. In Walker carcinosarcoma cells, the levels of the actin-membrane linker ezrin, a member of the ezrin-kexin-mosin (ERM) family, are increased on the opposite side of the bleb formation. It has been hypothesized that the membrane-cortex attachment asymmetry generated promotes bleb formation [31]. Moreover, focal intracellular pressure increases require local myosin activation [32], promoting cortical tearing and layering [33]. Hence, bleb formation is additionally dependent on the level of myosin contractility [8, 29].

### Bleb expansion

After initiation, intracellular pressure pushes cytosol into the bubbles for 5–30 s causing expansion [2]. The expansion rate and the size of the blebs depend on the biophysical properties of the cells [34] and the ability to perform cytoplasmic zoning. Cytoplasmic zoning, refers to the spatial organization and distribution of cellular components within the cytoplasm. This allows the cell to generate presser gradients through actin network reorganization [35, 36]. Hence, the localization of actinomyosin gel and the overall mechanical properties of a particular zone in the cell is altered, impacting the rate and extent of bleb expansion in the zone. Two models have been developed to describe the growth of blebs in cells. The Coarse-grained model utilizes macroscopic parameters such as pressure and tension to describe the mechanistic properties of the cell [8, 37, 38]. The actin cortex is modeled as actinomyosin gel that generates hydrostatic pressure in a pyroclastic cytoplasm. A higher bound of cortical tension is predicted to help initiate bleb expansion based on the reduced resistance of the plasma membrane against deformation. Below this threshold, cortical rupture and reduced membrane-cortex adhesion has no effect on bleb formation [39]. Additionally, the membrane source from which the bleb forms plays a role in this process [40]. The second approach is based on molecular models informed computational simulations that deduce cellular-scale behaviors from microscale processes [40–42]. However, these microscale models rely on a comprehensive understanding of molecular processes influencing cellular mechanics, information that is not always attainable through experimental means.

### Cortex repolymerization, retraction or extracellular vesicle release

Bleb retraction is the final stage of the blebbing process and involves the reconstruction of the actin cortex. At the end of bleb expansion, the actinomyosin cortex reassembles under the plasma membrane, and its contraction drives either bleb retreat or the formation of successive blebs [3]. In migrating cells, new blebs usually form shortly after the membrane underneath the cortex has repolymerized. This continuous expansion and retraction of blebs provide an explanation for the periodicity of pseudopod formation observed during amoeboid motility [43]. Alternatively, the protrusions of the cell membrane can pinch off to release extracellular vesicles (EVs) such as microvesicles and exosomes into the extracellular space. These vesicles transport bioactive molecules, including proteins, lipids, and nucleic acids, that can influence neighboring cells and contribute to peri-metastatic niche formation [44]. Moreover, the release of EVs through blebbing into the blood stream might facilitate the spread of cancer cells by enabling the transfer of oncogenic factors to distant sites, supporting pre metastatic niche formation [45, 46]. However, our knowledge of the connection between blebbing and EV release is still limited.

## Blebbing in cellular motility

Cell migration is crucial during embryonic development, wound healing, inflammation, cancer metastasis, and other pathological processes [47–50]. Actin-rich protrusions are preferentially formed at the leading edge of migration guiding cells. In cancer this is a prerequisite for blood or lymphatic vessels penetration. There are two primary types of cellular protrusions: some triggered by fluid pressure, including blebs, and those caused by actin polymerization, such lamellipodia, filopodia, invadopodia and podosomes [51, 52] (Fig. 2). Migration through blebbing was discovered in zebrafish primordial germ cells [3], the pathogen Entamoeba histolytica into the liver [29] and HEK-293 cells [53]. Non-invasive cells are converted to a preinvasive phenotype through the formation of blebs [54, 55]. In contrast, therapeutic bleb intervention with cytochalasin B or blebbistatin inhibits cell motility [56].

Several models have been formulated to explain how cell blebbing generates traction without relying on adhesion [57]. It was found that cells may establish connections with surrounding cells by applying lateral pushing forces during blebbing. In combination with the elongation of their leading edge, protrusions could lead to migration of the cell body [4, 34]. Cells encountering physical barriers or tight spaces can use blebbing as a mechanism to escape from confinement. The formation of blebs enables cells to squeeze through narrow openings [21]. This mechanism is used by Schwann cells, where blebs assist migration by interacting within the protrusions of adjacent cells [58]. In addition to locomotion, it was found that in motile zebrafish mesendodermal progenitor cells, blebs could also contribute to controlling cell migration precision [59]. Alba Diz-Muñoz and colleagues discovered that in a model mimicking zebrafish stomach development, modifying the ratio of actin-rich protrusions or blebbing sites caused an extension or contraction of the running phase, respectively. This contraction and elongation of the running phase resulted in an increased spatial dispersion of cells, thereby diminishing the precision of migration [59].

Nevertheless, the interplay between blebbing migration and metabolic stress, particularly hypoxia, remains less clear. While the effects of mechanical stress on blebbing are well documented, the specific impact of hypoxia on blebbing needs further exploration. It is known that hypoxic conditions, prevalent in tumor microenvironments, enforces EMT. Hypoxia impairs cell adhesion and enhance motility, which contributes to increased tumor invasiveness [60]. Notably, the calcium-dependent proteases of the calpain family, significantly influence tumor cell migration and invasion under hypoxic conditions [61]. Recent discoveries showed that hypoxia triggers metabolic shifts that enhance blebbing in cancer cells leading to cell invasion [60]. These shifts involve increased glycolysis and altered energy production, which destabilize the actin cytoskeleton. Specifically, HIF-1α promotes bleb formation and supports tumor cell migration via Calpain-2-induced actin depolymerization. This depolymerization destabilizes the actin cortex and facilitates enhanced cell motility through dense extracellular matrices [62]. All told, this research suggest that a metabolic alterations reside upstream of blebbing migration with possible feedback mechanisms after reaching metabolic equilibrium [63].

In summary, blebbing is a versatile cellular process that contributes to spontaneous cell migration, response to environmental cues, and adaptation to different microenvironments. The dynamic nature of blebbing allows cells to navigate through complex and challenging conditions. In addition to their role in cell motility, recent studies found that they are also involved in regulating tumor migration and invasion, in M2 melanoma and Walker cancer cells [64, 65] as well as breast cancer cells [66].

## Blebbing in tumor invasion and metastasis

Limitations in the understanding of pathological migration and infiltration of metastatic cells are critical bottlenecks in our comprehension of cancer morbidity. The main migratory mechanisms described in cancer are mesenchymal and amoeboid (blebbing) based [67]. Mesenchymal migration involves the formation of filopodia and lamellipodia at the leading edge of cells, driven by actin polymerization. Considerable reorganization of the extracellular matrix (ECM) and strong integrin adhesion are observed in these cancers [68, 69]. Amoeboid migration patterns refer to cells that exhibit a predominantly rounded morphology, named after the morphological similarity of these cells to those of amoebas [70, 71]. They navigate through narrow spaces using robust actomyosin contractility without the need for extracellular matrix (ECM) adhesion, restructuring, or proteolytic degradation. Amoeboid based migration is an intriguing marker candidate of metastatic cancer, as it is the only mechanism possible for extravasation (Fig. 1).

The tumor microenvironment generally acidifies the surrounding tissue, which supports tumor development [72]. However, recent research has revealed that a brief (5-minute) exposure of endocrine-resistant breast cancer cells to an alkaline pH environment induces cell rounding and contraction. Moreover, the formation of membrane blebs rich in actin on the outer membrane of the cells, not associated with cell apoptosis, was described [73]. In an alkaline environment, key molecules associated with cell motility and invasion, such as integrin α2, JAM-1 (junctional adhesion molecule-1), and FAK (focal adhesion kinase), undergo translocation into the cytoplasmic compartment of the newly formed blebs [49, 73]. Interestingly, molecules unrelated to motility, such as vimentin, do not exhibit cytoplasmic redistribution in response to alkaline pH. Blebbing is traditionally believed to be inhibited during the execution phase of apoptosis [4]. However, apoptotic and non-apoptotic blebs coexist in the same cell, depending on the duration of alkaline exposure. Specifically, a brief exposure to alkaline pH, increased the motility and invasive capability in endocrine-resistant cancer cells through blebbing. Fast restoration of physiological pH levels reverses this blebbing, while prolonged exposure (beyond 4 h) leads to apoptosis [74]. Alkaline-induced blebbing in Estrogen receptor negative breast cancer cells reduces the epithelial marker E-cadherin supporting epithelial to mesenchymal transition. E-cadherin reduction weakens cell-cell contacts and increases morphological flexibility [22]. Moreover, the cytoplasmic composition of the newly formed blebs differs from the rest of the cell, concentrating molecules critical for cell motility and invasion.

Unlike endocrine-resistant breast cancer cells, Kato et al. found that certain melanoma cell lines exhibit enhanced invasive capabilities under acidic pH conditions, suggesting cancer-specific pH effects [75]. Orgaz JL et al. explored the development of targeted and immune checkpoint blockade-based therapy resistance in melanoma and the role of cytoskeletal remodeling [24]. Therapy-resistant melanoma undergo cytoskeletal remodeling, leading to increased levels of myosin II, essential for the survival of targeted and immunotherapy-resistant cells. Hence, targeting the ROCK-myosin II pathway could be a potential therapeutic strategy to overcome resistance in melanoma [20]. Cantelli G et al. investigated how TGF-β signaling regulates melanoma migration independently of epithelial-mesenchymal transition (EMT) [23]. TGF-β upregulated the contractile actomyosin cytoskeleton in melanoma through SMAD/CITED1-driven transcription. CITED1, an adaptor protein, is involved in melanocyte pigmentation and is upregulated in certain cancers. Targeting CITED1 is a potential therapeutic strategy for melanoma [19]. Weems AD et al. focused on the role of bleb formation and septin signaling in NRAS-mutant metastatic melanoma. Blebbing resulted in septin signaling hubs, which improved the survival of cancer cells in low-adhesion environments [5]. This bleb-septin signaling pathway is not unique to melanoma but is a conserved pathway in both mice and humans. This indicates that targeting septins is a potential strategy for treating metastatic melanoma [5]. Overall, these studies provide insights into different aspects of melanoma development and therapy resistance, highlighting novel targets and strategies for this aggressive cancer.

Khan ZS et al. used microfluidic and hypo-osmotic methods to observe biophysical effectors of blebbing [76]. Reduced blebbing decreased metastatic potential. Moreover, they examined biomechanical effectors of blebbing using a cell rheology model. Low cellular stiffness was associated with increased cell blebbing in prostate cancer. Hence, blebbing and cell stiffness could serve as biomechanical markers for metastatic prostate cancer [76]. Counterintuitively, myosin II does not play a key role in prostate cancer blebbing, and ezrin and myosin expression had no effect on blebbing in metastatic prostate cancer cells. They propose microfluidics to directly detect blebbing in metastatic prostate cancer as a simple and inexpensive clinical tool. Overall, this research provides insights into the relationship between blebbing, cell stiffness, and metastatic potential in prostate cancer cells, and suggests diagnostic applications for microfluidics in detecting metastatic cancer cells.

Phospholipase D (PLD1 & 2) activity increases blebbing in Fibrosarcoma. The small molecule inhibitor 5-fluoro-2-indolyl des-chlorohalopemide (FIPI), blocks PLD1 and PLD2 activity, effectively suppressing bleb formation in the HT1080 Fibrosarcoma cell line [77]. In addition, this study revealed that the downstream products of PLD2, phosphatidic acid, lysophosphatidic acid, and phosphatidylinositol 4,5-bisphosphate (PIP2), also play important roles in cell blebbing. It is notable to mention that changes in PIP2 levels affected the size but not the number of blebs formed [77].

## Potential anti-blebbing drugs for cancer therapy

**Fig. 4:**
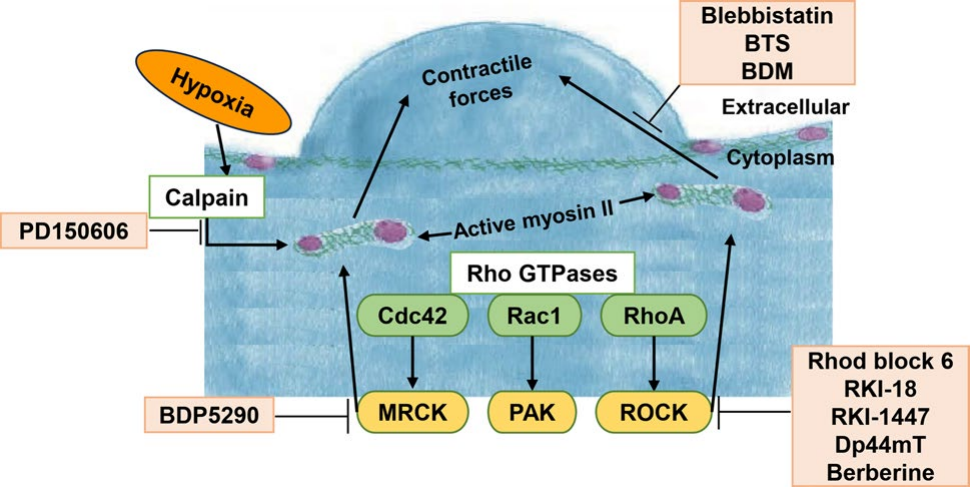
Regulation of cell blebbing by signaling pathways and specific inhibitors. The key signaling pathways involved in the regulation of cell blebbing is driven by actomyosin cytoskeletal contraction. The Rho family GTPases, including Cdc42, Rac1, and RhoA, are pivotal regulators activating downstream kinases MRCK, PAK, and ROCK, respectively. These kinases promote the activation of myosin II, which generates the contractile forces necessary for bleb formation at the cell surface. Alternatively, blebbing is introduced by the hypoxia sensitive kinase Calpain. Pharmacological inhibitors are shown in light orange boxes

As introduced, cellular blebbing is dependent on the activation of myosin II (NMII). NMII consists of heavy chains: a globular head structure that binds actin and adenosine triphosphate (ATP) [78, 79], a neck region that binds both the essential and regulatory light chains (ELC and RLC) [80], and a tail region that homodimerizes in a helical fashion [81]. NMII has an important role in driving tumor invasion and metastasis [82]. Blocking bleb formation may selectively inhibit metastatic cancer migration. Multiple inhibitors target NMII and blebbing in general (Fig. 4; Table 1). This facilitates blebbing therapy additionally to chemotherapeutic therapy.

**Table 1:** Potential therapeutic drugs targeting blebbing for clinical applications

Inhibitor name	Structural formula	Primary myosin target	Clinical phase	References
Blebbistatin		Myosin II (nonmuscle, skeletal muscle)	Experimental	[70]
N-benzyl-p-toluene sulphonamide (BTS)		Myosin II (fast-twitch skeletal muscle)	Experimental	[71, 72]
2,3-Butanedione monoxime (BDM)		Myosin II (skeletal muscle)	Experimental	[73, 74]
BDP5290		MRCK inhibitor	Experimental	[75]
Rhod block 6		ROCK inhibitor	Experimental	[76]
RKI-18		ROCK inhibitor	Experimental	[77]
RKI-1447		ROCK inhibitor	Experimental	[78]
Di-2-pyridylketone 4,4-dimethyl-3-thiosemicarbazone (Dp44mT)		ROCK and RLC inhibitor	Experimental	[79]
Berberine		RhoA/ROCK inhibitor	Phase I	[80, 81]
PD150606		Calpain inhibitor	Experimental	[60]

Blebbistatin is a widely used myosin-specific small molecule inhibitor. This small molecule derivative of 1-phenyl-2-pyrrolidone can permeate cell membranes [83], and blocks myosin II function by inhibiting an essential ATPase cycle (Fig. 4). It exhibits noncompetitive binding to the hydrophobic pocket at the apex of the 50-kDa cleft in the myosin II motor domain. The cleft opens and closes during the contraction cycle [84]. Specifically, blebbistatin stabilizes myosin in its metastable or transition state, before the force generation step upon recombination of myosin with actin. Blebbistatin binds and stabilizes the closed ADP/Pi (hydrolysis product of ATP) bound myosin II intermediate state, thereby preventing the release of Pi and the associated myosin power stroke [85]. Blebbistatin has little to no impact on smooth muscle myosin II and class I, V, and X myosin, but it is an effective inhibitor of skeletal and non-muscle myosin II isoforms [86]. This prevents the formation of strongly bound non-functional myosin complexes with other proteins that can have severe side effects [85].

N-benzyl-p-toluene sulfonamide (BTS) was identified in a small molecule inhibitor screen for skeletal muscle myosin II [87]. BTS inhibits actin activation and suppresses ATPase activity. This impedes the gliding movement of myosin II in skeletal muscle in-vitro (Fig. 4). In-vivo, BTS transmits through the cell membrane and blocks contraction, inhibiting skeletal muscle fibers synthesis [88]. Analyzing the kinetics of the myosin II ATPase cycle, BTS reduced the rate of inorganic phosphate (Pi) release from myosin II ATPase by more than 100-fold in the presence of actin. The rate of ADP separation decreased which consequently prevented the release of Pi. Subsequently, it was found that myosin II-ADP-Pi intermediates stabilized by BTS had reduced affinity for actin. This suggests that BTS acts as an inhibitor of muscle contraction in-vivo by blocking Pi release and actin binding [89]. BTS selectively binds fast skeletal muscle myosin II non-competitively without inducing calcium transients. In addition, the inhibition is reversible and does not significantly affect myosin or actin expression. Hence, BTS might be an effective medicine to inhibit tumor cell blebbing. However, further investigation is needed to elucidate the effects of BTS on other myosin classes.

2,3-Butanedione monoxime (BDM) is a non-competitive small molecule inhibitor of myosin II, like BTS and blebbistatin (Fig. 4). Although it has a lower affinity for myosin [90], myosin II function is blocked by impeding Pi release and stabilizing the myosin-ADP-Pi intermediate [91, 92]. BDM was initially designed as an acetylcholinesterase reactivator [93], but it was discovered to be a broad inhibitor for skeletal muscle myosin II [94], non-muscle myosin II, myosin V, and myosin X [95]. BDM showed off target effects on a wide range of proteins and molecular mechanisms without requiring the activation of myosin ATPase [96, 97]. Therefore, BDM might be of limited use in targeting blebbing in tumor cells.

Moreover, NMII downstream inhibitors block cellular blebbing. Serine/threonine kinases are downstream of NMII. They phosphorylate myosin RLC on residues Thr18 and Ser19, controlling the activity of NMII [98]. These kinases promote Rho GTPase downstream signaling after binding GTP (p21-associated kinase (PAK), myotonic dystrophy kinase-related Cdc42-binding kinase (MRCK), RhoA-associated kinase (ROCK) and citron kinase [99–102] (Fig. 4). Therefore, the MRCK inhibitor BDP5290, can be considered a candidate drug to inhibit cellular blebbing. In addition, ROCK inhibitors showed significant anti-tumor metastatic effects in preclinical research [103, 104]. ROCK inhibitors such as Rhod block 6, RKI-18, RKI-1447, Di-2-pyridylketone 4,4-dimethyl-3-thiosemicarbazone (Dp44mT) and Berberine [105–109] are expected to be among the most promising therapeutic agents to inhibit metastasis of tumor and tumor growth. ROCK targeting is interesting for cancer therapy, with suppressing effects on tumor cell migration and invasion, angiogenesis, and inducing tumor cell apoptosis (Fig. 4). However, the use of ROCK inhibitors may also lead to side effects, such as cytotoxicity in normal cells, cardiovascular effects, gastrointestinal discomfort, and other adverse reactions [110, 111]. Therefore, it is necessary to carefully balance their potential benefits and adverse effects, in clinical applications monitoring patient safety and treatment efficacy accordingly.

Given the relationship between hypoxia and migration via calpain proteases (Fig. 4), PD150606, a calpain inhibitor, shows notable therapeutic potential [62]. Calpain inhibition prevents the transition of tumor cells from an elongated to a rounded morphology, a requirement for aggressive tumor cell invasion [112]. Additionally, it reverses the changes such as actin cytoskeleton destabilization, loss of cell adhesion, and altered motility, caused by hypoxia, thereby maintaining cell adhesion and normalizing cell motility [62]. Further research is needed to elaborate the influence of Calpain inhibition on cellular blebbing including possible beneficial off target effects and in-vivo application of Calpain inhibitors.

## Conclusions and future directions

Tumor cells employ the biological phenomenon of blebbing as a mechanism to infiltrate adjacent tissues and intravasate/extravasate the bloodstream, crucial steps in the metastatic cascade [5, 113]. This intricate process is underpinned by cytoskeletal reorganization mediated by alterations in the dynamics of actin and myosin. Blebbing enhances tumor cell survival in the bloodstream, where it provides resilience against shear forces and mitigates apoptosis triggered by the loss of cell-matrix interactions [5]. Notably, elevated levels of blebbing have been correlated with aggressive cancer phenotypes and unfavorable clinical outcomes, positioning it as pro-metastatic marker for specific cancers. Khan et al. suggested microfluidic techniques offer a streamlined and cost-efficient approach to diagnosing tumor cell blebbing [76]. Despite its potential, microfluidic technology encounters hurdles in effectively detecting tumor biomarkers. These challenges include inadequate sensitivity and specificity, intricate sample preprocessing requirements, limited experiment optimization and standardization, constrained detection range, and elevated costs for establishing the technology. Addressing these limitations requires further attention and innovation before the establishment as routine practice in the clinic [114].

A significant body of evidence suggests that blebbing is widely present in the process of tumor metastasis, so inhibition of blebbing could specifically block metastasis [5, 74, 76]. Although this idea has gradually been accepted, targeted drugs against blebbing are limited due to the lack of an accurate understanding of the regulation mechanism. Blebbistatin, BTS and BDM, these three pharmaceutical agents each offer distinctive merits and drawbacks in managing tumor cell blebbing. Blebbistatin and BTS exhibit notable efficacy in suppressing cell blebbing, potentially mitigating tumor cell invasion and metastasis, albeit necessitating further rigorous evaluation for long-term safety and clinical applicability [83, 87, 89]. Conversely, while BDM demonstrates cytotoxicity in specific experimental contexts, its efficacy in blebbing inhibition appears comparatively modest, warranting comprehensive investigation into its translational potential and safety profile [91, 94]. Hence, the judicious selection of therapeutic interventions for tumor cell blebbing mandates a nuanced consideration of their efficacy, safety, and translational feasibility. Rhod block 6 [105], RKI-18 [106], RKI-1447 [107], Dp44mT [108], and Berberine [109, 115] have exhibited promising efficacy in experimental in-vitro models by attenuating cell blebbing, thereby offering auspicious avenues for combating tumor metastasis. Nevertheless, comprehensive validation through additional experimental in-vivo safety and efficacy data is imperative prior to their translation into clinical applications. The available basic studies impose critical limitations on clinical transferability of these drugs.

We established that dynamic alterations observed in tumor cell blebbing exert profound effects on tumor cell motility, invasiveness, and metastatic potential. Therapeutic interventions targeting blebbing offer a promising avenue for impeding tumor metastasis with minimal impact on normal cellular proliferation, thus representing a novel strategy with limited treatment-associated adverse effects. Moving forward, research and clinical endeavors encompass several pivotal dimensions: Firstly, despite the discovery of potential blebbing inhibitors, ongoing chemical exploration is essential. Novel compounds with heightened efficacy and specificity for mitigating tumor cell blebbing are needed. Drug screening platforms, molecular design strategies, and rigorous in-vitro evaluations have to be employed to identify promising new drug candidates. Secondly, quantifying blebbing experimentally poses challenges owing to its dynamic morphological nature. Developing robust tools and methodologies to accurately gauge blebbing activity is crucial. Informed by Weems AD et al. [5], combining cell deformation markers, cell tracking and fast, high-resolution imaging will provide reliable blebbing assessment in-vitro. Thirdly, elucidating the optimal timing and dosing for drug administration remains a critical enigma. While certain drugs exhibit blebbing inhibition, defining the ideal treatment window is critical. Experimental in-vivo experiments investigating therapeutic efficacy, safety and dosage are needed. Lastly, the inherent heterogeneity within primary tumors engenders diverse blebbing activities across different cells. To address this, histological makers for lamellipodia dependent migrations are needed to classify this heterogeneity and determine its migratory capacity.

In essence, the profound insights from tumor cell blebbing research imposes a paradigm shift, offering a novel aspect in understanding metastatic migration. However, translating these insights into clinical practice remains far away. Never the less, recent discoveries poise significant knowledge in the fundamentals of tumor bleb formation [5]. Hence, we are confident blebbing will be a cornerstone of future metastatic therapeutics and diagnostics.

## Data Availability

No datasets were generated or analysed during the current study.

## References

[1] Hagmann J, Burger MM, Dagan D (1999) Regulation of plasma membrane blebbing by the cytoskeleton. J Cell Biochem 73:488–499 10733343

[2] Charras GT (2008) A short history of blebbing. J Microsc 231:466–478 18755002 10.1111/j.1365-2818.2008.02059.x

[3] Blaser H, Reichman-Fried M, Castanon I et al (2006) Migration of zebrafish primordial germ cells: a role for myosin contraction and cytoplasmic flow. Dev Cell 11:613–627 17084355 10.1016/j.devcel.2006.09.023

[4] Charras G, Paluch E (2008) Blebs lead the way: how to migrate without lamellipodia. Nat Rev Mol Cell Biol 9:730–736 18628785 10.1038/nrm2453

[5] Weems AD, Welf ES, Driscoll MK et al (2023) Blebs promote cell survival by assembling oncogenic signalling hubs. Nature 615:517–525 36859545 10.1038/s41586-023-05758-6 PMC10881276

[6] Hogue MJ (1919) The effect of hypotonic and hypotonic solutions on fibroblasts of the embryonic chick heart in vitro. J Exp Med 30:617–648. 10.1084/jem.30.6.617 19868382 10.1084/jem.30.6.617 PMC2126669

[7] Mills JC, Stone NL, Erhardt J, Pittman RN (1998) Apoptotic membrane blebbing is regulated by myosin light chain phosphorylation. J Cell Biol 140:627–636 9456322 10.1083/jcb.140.3.627 PMC2140178

[8] Norman LL, Brugés J, Sengupta K et al (2010) Cell blebbing and membrane area homeostasis in spreading and retracting cells. Biophys J 99:1726–1733. 10.1016/j.bpj.2010.07.031 20858416 10.1016/j.bpj.2010.07.031 PMC2944031

[9] Norman L, Sengupta K, Aranda-Espinoza H (2011) Blebbing dynamics during endothelial cell spreading. Eur J Cell Biol 90:37–48. 10.1016/j.ejcb.2010.09.013 21087809 10.1016/j.ejcb.2010.09.013

[10] Fackler OT, Grosse R (2008) Cell motility through plasma membrane blebbing. J Cell Biol 181:879–884. 10.1083/jcb.200802081 18541702 10.1083/jcb.200802081 PMC2426937

[11] Svitkina TM, Verkhovsky AB, McQuade KM, Borisy GG (1997) Analysis of the actin-myosin II system in fish epidermal keratocytes: mechanism of cell body translocation. J Cell Biol 139:397–415. 10.1083/jcb.139.2.397 9334344 10.1083/jcb.139.2.397 PMC2139803

[12] Abercrombie M, Heaysman JEM, Pegrum SM (1970) The locomotion of fibroblasts in culture I. movements of the leading edge. Exp Cell Res 59:393–398. 10.1016/0014-4827(70)90646-4 4907703 10.1016/0014-4827(70)90646-4

[13] Lämmermann T, Sixt M (2009) Mechanical modes of amoeboid cell migration. Curr Opin Cell Biol 21:636–644. 10.1016/j.ceb.2009.05.003 19523798 10.1016/j.ceb.2009.05.003

[14] Friedl P (2004) Prespecification and plasticity: shifting mechanisms of cell migration. Curr Opin Cell Biol 16:14–23. 10.1016/j.ceb.2003.11.001 15037300 10.1016/j.ceb.2003.11.001

[15] Neophytou CM, Panagi M, Stylianopoulos T, Papageorgis P (2021) The role of tumor microenvironment in cancer metastasis: molecular mechanisms and therapeutic opportunities. Cancers (Basel). 10.3390/cancers13092053 33922795 10.3390/cancers13092053 PMC8122975

[16] Fares J, Fares MY, Khachfe HH et al (2020) Molecular principles of metastasis: a hallmark of cancer revisited. Sig Transduct Target Ther 5:1–17. 10.1038/s41392-020-0134-x 10.1038/s41392-020-0134-x PMC7067809 32296047

[17] Novikov NM, Zolotaryova SY, Gautreau AM, Denisov EV (2021) Mutational drivers of cancer cell migration and invasion. Br J Cancer 124:102–114. 10.1038/s41416-020-01149-0 33204027 10.1038/s41416-020-01149-0 PMC7784720

[18] Rottner K, Schaks M (2019) Assembling actin filaments for protrusion. Curr Opin Cell Biol 56:53–63. 10.1016/j.ceb.2018.09.004 30278304 10.1016/j.ceb.2018.09.004

[19] Fritz-Laylin LK, Lord SJ, Kakley M, Mullins RD (2018) Concise language promotes clear thinking about cell shape and locomotion. BioEssays 40:e1700225. 10.1002/bies.201700225 29846958 10.1002/bies.201700225 PMC6175535

[20] Charras GT, Hu C-K, Coughlin M, Mitchison TJ (2006) Reassembly of contractile actin cortex in cell blebs. J Cell Biol 175:477–490. 10.1083/jcb.200602085 17088428 10.1083/jcb.200602085 PMC2064524

[21] Tozluoğlu M, Tournier AL, Jenkins RP et al (2013) Matrix geometry determines optimal cancer cell migration strategy and modulates response to interventions. Nat Cell Biol 15:751–762. 10.1038/ncb2775 23792690 10.1038/ncb2775

[22] Guzman A, Avard RC, Devanny AJ et al (2020) Delineating the role of membrane blebs in a hybrid mode of cancer cell invasion in three-dimensional environments. J Cell Sci 133:jcs236778. 10.1242/jcs.236778 32193332 10.1242/jcs.236778 PMC7197870

[23] Cantelli G, Orgaz JL, Rodriguez-Hernandez I et al (2015) TGF-β-induced transcription sustains amoeboid melanoma migration and dissemination. Curr Biol 25:2899–2914. 10.1016/j.cub.2015.09.054 26526369 10.1016/j.cub.2015.09.054 PMC4651903

[24] Orgaz JL, Crosas-Molist E, Sadok A et al (2020) Myosin II reactivation and cytoskeletal remodeling as a hallmark and a vulnerability in melanoma therapy resistance. Cancer Cell 37:85-103e9. 10.1016/j.ccell.2019.12.003 31935375 10.1016/j.ccell.2019.12.003 PMC6958528

[25] Sanz-Moreno V, Gaggioli C, Yeo M et al (2011) ROCK and JAK1 signaling cooperate to control actomyosin contractility in tumor cells and stroma. Cancer Cell 20:229–245. 10.1016/j.ccr.2011.06.018 21840487 10.1016/j.ccr.2011.06.018

[26] Cunningham CC (1995) Actin polymerization and intracellular solvent flow in cell surface blebbing. J Cell Biol 129:1589–1599 7790356 10.1083/jcb.129.6.1589 PMC2291187

[27] Keller H, Eggli P (1998) Protrusive activity, cytoplasmic compartmentalization, and restriction rings in locomoting blebbing Walker carcinosarcoma cells are related to detachment of cortical actin from the plasma membrane. Cell Motil Cytoskeleton 41:181–193. 9786092 10.1002/(SICI)1097-0169(1998)41:2<181::AID-CM8>3.0.CO;2-H

[28] Rentsch PS, Keller H (2000) Suction pressure can induce uncoupling of the plasma membrane from cortical actin. Eur J Cell Biol 79:975–981. 10.1078/0171-9335-00124 11152288 10.1078/0171-9335-00124

[29] Maugis B, Brugués J, Nassoy P et al (2010) Dynamic instability of the intracellular pressure drives bleb-based motility. J Cell Sci 123:3884–3892. 10.1242/jcs.065672 20980385 10.1242/jcs.065672

[30] Sedzinski J, Biro M, Oswald A et al (2011) Polar actomyosin contractility destabilizes the position of the cytokinetic furrow. Nature 476:462–466. 10.1038/nature10286 21822289 10.1038/nature10286

[31] Martinelli S, Chen EJH, Clarke F et al (2013) Ezrin/Radixin/Moesin proteins and flotillins cooperate to promote uropod formation in T cells. Front Immunol 4:84. 10.3389/fimmu.2013.00084 23579783 10.3389/fimmu.2013.00084 PMC3619129

[32] Charras GT, Yarrow JC, Horton MA et al (2005) Non-equilibration of hydrostatic pressure in blebbing cells. Nature 435:365–369. 10.1038/nature03550 15902261 10.1038/nature03550 PMC1564437

[33] Paluch E, Piel M, Prost J et al (2005) Cortical actomyosin breakage triggers shape oscillations in cells and cell fragments. Biophys J 89:724–733. 10.1529/biophysj.105.060590 15879479 10.1529/biophysj.105.060590 PMC1366569

[34] Paluch EK, Raz E (2013) The role and regulation of blebs in cell migration. Curr Opin Cell Biol 25:582–590. 10.1016/j.ceb.2013.05.005 23786923 10.1016/j.ceb.2013.05.005 PMC3989058

[35] Fujii Y, Ikenouchi J (2024) Cytoplasmic zoning in membrane blebs. J Biochem 175:133–140. 10.1093/jb/mvad084 37943501 10.1093/jb/mvad084

[36] McLane LT, Chang P, Granqvist A et al (2013) Spatial organization and mechanical properties of the pericellular matrix on chondrocytes. Biophys J 104:986–996. 10.1016/j.bpj.2013.01.028 23473481 10.1016/j.bpj.2013.01.028 PMC3870807

[37] Lim FY, Chiam K-H, Mahadevan L (2012) The size, shape, and dynamics of cellular blebs. EPL 100:28004. 10.1209/0295-5075/100/28004

[38] Boulbitch A, Simson R, Simson DA et al (2000) Shape instability of a biomembrane driven by a local softening of the underlying actin cortex. Phys Rev E Stat Phys Plasmas Fluids Relat Interdiscip Top 62:3974–3985. 10.1103/physreve.62.3974 10.1103/physreve.62.3974 11088918

[39] Tinevez J-Y, Schulze U, Salbreux G et al (2009) Role of cortical tension in bleb growth. Proc Natl Acad Sci U S A 106:18581–18586. 10.1073/pnas.0903353106 19846787 10.1073/pnas.0903353106 PMC2765453

[40] Young J, Mitran S (2010) A numerical model of cellular blebbing: a volume-conserving, fluid-structure interaction model of the entire cell. J Biomech 43:210. 10.1016/j.jbiomech.2009.09.025 19875121 10.1016/j.jbiomech.2009.09.025 PMC2813352

[41] Spangler EJ, Harvey CW, Revalee JD et al (2011) Computer simulation of cytoskeleton-induced blebbing in lipid membranes. Phys Rev E Stat Nonlin Soft Matter Phys 84:051906. 10.1103/PhysRevE.84.051906 22181443 10.1103/PhysRevE.84.051906

[42] Strychalski W, Guy RD (2013) A computational model of bleb formation. Math Med Biol 30:115–130. 10.1093/imammb/dqr030 22294562 10.1093/imammb/dqr030 PMC4104658

[43] Décave E, Rieu D, Dalous J et al (2003) Shear flow-induced motility of *Dictyostelium discoideum* cells on solid substrate. J Cell Sci 116:4331–4343. 10.1242/jcs.00726 12966168 10.1242/jcs.00726

[44] Takahashi Y, Takakura Y (2023) Extracellular vesicle-based therapeutics: extracellular vesicles as therapeutic targets and agents. Pharmacol Ther 242:108352. 10.1016/j.pharmthera.2023.108352 36702209 10.1016/j.pharmthera.2023.108352

[45] Chang W-H, Cerione RA, Antonyak MA (2021) Extracellular vesicles and their roles in cancer progression. Methods Mol Biol 2174:143–170. 10.1007/978-1-0716-0759-6_10 32813249 10.1007/978-1-0716-0759-6_10 PMC8008708

[46] Geissler M, Jia W, Kiraz EN et al (2023) The brain pre-metastatic niche: biological and technical advancements. Int J Mol Sci 24:10055. 10.3390/ijms241210055 37373202 10.3390/ijms241210055 PMC10298140

[47] Pollard TD, Borisy GG (2003) Cellular motility driven by assembly and disassembly of actin filaments. Cell 112:453–465. 10.1016/s0092-8674(03)00120-x 12600310 10.1016/s0092-8674(03)00120-x

[48] Torgerson RR, McNiven MA (1998) The actin-myosin cytoskeleton mediates reversible agonist-induced membrane blebbing. J Cell Sci 111(Pt 19):2911–2922. 10.1242/jcs.111.19.2911 9730983 10.1242/jcs.111.19.2911

[49] Khajah MA, Almohri I, Mathew PM, Luqmani YA (2013) Extracellular alkaline pH leads to increased metastatic potential of estrogen receptor silenced endocrine resistant breast cancer cells. PLoS ONE 8:e76327. 10.1371/journal.pone.0076327 24098477 10.1371/journal.pone.0076327 PMC3788134

[40] Loitto V-M, Forslund T, Sundqvist T, Magnusson K-E, Gustafsson M (2002) Neutrophil leukocyte motility requires directed water influx. J Leukoc Biol 71:212–222. 10.1189/jlb.71.2.212 11818441

[51] Wolf K, Mazo I, Leung H et al (2003) Compensation mechanism in tumor cell migration: mesenchymal-amoeboid transition after blocking of pericellular proteolysis. J Cell Biol 160:267–277. 10.1083/jcb.200209006 12527751 10.1083/jcb.200209006 PMC2172637

[52] Alblazi KMO, Siar CH (2015) Cellular protrusions–lamellipodia, filopodia, invadopodia and podosomes–and their roles in progression of orofacial tumours: current understanding. Asian Pac J Cancer Prev 16:2187–2191. 10.7314/apjcp.2015.16.6.2187 25824735 10.7314/apjcp.2015.16.6.2187

[53] Karlsson T, Bolshakova A, Magalhães MAO et al (2013) Fluxes of water through aquaporin 9 weaken membrane-cytoskeleton anchorage and promote formation of membrane protrusions. PLoS ONE 8:e59901. 10.1371/journal.pone.0059901 23573219 10.1371/journal.pone.0059901 PMC3616121

[54] Tournaviti S, Hannemann S, Terjung S et al (2007) SH4-domain-induced plasma membrane dynamization promotes bleb-associated cell motility. J Cell Sci 120:3820–3829. 10.1242/jcs.011130 17959630 10.1242/jcs.011130

[55] Gadea G, de Toledo M, Anguille C, Roux P (2007) Loss of p53 promotes RhoA–ROCK-dependent cell migration and invasion in 3D matrices. J Cell Biol 178:23–30. 10.1083/jcb.200701120 17606864 10.1083/jcb.200701120 PMC2064414

[56] D’andrea-Winslow L, Novitski AK (2008) Active bleb formation is abated in *Lytechinus variegatus* red spherule coelomocytes after disruption of acto-myosin contractility. Integr Zool 3:115–122. 10.1111/j.1749-4877.2008.00086.x 21396059 10.1111/j.1749-4877.2008.00086.x

[57] Schick J, Raz E (2022) Blebs—formation, regulation, positioning, and role in amoeboid cell migration. Front Cell Dev Biol 10:926394. 10.3389/fcell.2022.926394 35912094 10.3389/fcell.2022.926394 PMC9337749

[58] Cattin A-L, Burden JJ, Van Emmenis L et al (2015) Macrophage-induced blood vessels guide Schwann cell-mediated regeneration of peripheral nerves. Cell 162:1127–1139. 10.1016/j.cell.2015.07.021 26279190 10.1016/j.cell.2015.07.021 PMC4553238

[59] Diz-Muñoz A, Romanczuk P, Yu W et al (2016) Steering cell migration by alternating blebs and actin-rich protrusions. BMC Biol 14:74. 10.1186/s12915-016-0294-x 27589901 10.1186/s12915-016-0294-x PMC5010735

[60] Ildiz ES, Gvozdenovic A, Kovacs WJ, Aceto N (2023) Travelling under pressure - hypoxia and shear stress in the metastatic journey. Clin Exp Metastasis 40:375–394. 10.1007/s10585-023-10224-8 37490147 10.1007/s10585-023-10224-8 PMC10495280

[61] Miyoshi H, Umeshita K, Sakon M et al (1996) Calpain activation in plasma membrane bleb formation during tert-butyl hydroperoxide-induced rat hepatocyte injury. Gastroenterology 110:1897–1904. 10.1053/gast.1996.v110.pm8964416 8964416 10.1053/gast.1996.v110.pm8964416

[62] Boekhorst Vte, Jiang L, Mählen M et al (2022) Calpain-2 regulates hypoxia/HIF-induced plasticity toward amoeboid cancer cell migration and metastasis. Curr Biol 32:412–427e8. 34883047 10.1016/j.cub.2021.11.040 PMC10439789

[63] Chen Z, Han F, Du Y et al (2023) Hypoxic microenvironment in cancer: molecular mechanisms and therapeutic interventions. Sig Transduct Target Ther 8:1–23. 10.1038/s41392-023-01332-8 10.1038/s41392-023-01332-8 PMC9935926 36797231

[64] Cunningham CC, Gorlin JB, Kwiatkowski DJ et al (1992) Actin-binding protein requirement for cortical stability and efficient locomotion. Science 255:325–327. 10.1126/science.1549777 1549777 10.1126/science.1549777

[65] Keller Hu, Bebie H (1996) Protrusive activity quantitatively determines the rate and direction of cell locomotion. Cell Motil 33:241–251. 10.1002/(SICI)1097-0169(1996)33:4<241::AID-CM1>3.0.CO;2-C 8801030

[66] Laser-Azogui A, Diamant-Levi T, Israeli S et al (2014) Met-induced membrane blebbing leads to amoeboid cell motility and invasion. Oncogene 33:1788–1798. 10.1038/onc.2013.138 23665680 10.1038/onc.2013.138

[67] Clark AG, Vignjevic DM (2015) Modes of cancer cell invasion and the role of the microenvironment. Curr Opin Cell Biol 36:13–22. 10.1016/j.ceb.2015.06.004 26183445 10.1016/j.ceb.2015.06.004

[68] Friedl P, Wolf K (2010) Plasticity of cell migration: a multiscale tuning model. J Cell Biol 188:11–19. 10.1083/jcb.200909003 19951899 10.1083/jcb.200909003 PMC2812848

[69] Gardel ML, Schneider IC, Aratyn-Schaus Y, Waterman CM (2010) Mechanical integration of actin and adhesion dynamics in cell migration. Annu Rev Cell Dev Biol 26:315–333. 10.1146/annurev.cellbio.011209.122036 19575647 10.1146/annurev.cellbio.011209.122036 PMC4437624

[70] Yamada KM, Sixt M (2019) Mechanisms of 3D cell migration. Nat Rev Mol Cell Biol 20:738–752. 10.1038/s41580-019-0172-9 31582855 10.1038/s41580-019-0172-9

[71] Bergert M, Chandradoss SD, Desai RA, Paluch E (2012) Cell mechanics control rapid transitions between blebs and lamellipodia during migration. Proc Natl Acad Sci U S A 109:14434–14439. 10.1073/pnas.1207968109 22786929 10.1073/pnas.1207968109 PMC3437886

[72] Estrella V, Chen T, Lloyd M et al (2013) Acidity generated by the tumor microenvironment drives local invasion. Cancer Res 73:1524–1535. 10.1158/0008-5472.CAN-12-2796 23288510 10.1158/0008-5472.CAN-12-2796 PMC3594450

[73] Khajah MA, Mathew PM, Alam-Eldin NS, Luqmani YA (2015) Bleb formation is induced by alkaline but not acidic pH in estrogen receptor silenced breast cancer cells. Int J Oncol 46:1685–1698. 10.3892/ijo.2015.2884 25672508 10.3892/ijo.2015.2884

[74] Khajah MA, Luqmani YA (2016) Involvement of membrane blebbing in immunological disorders and cancer. Med Princ Pract 25(Suppl 2):18–27. 10.1159/000441848 26488882 10.1159/000441848 PMC5588526

[75] Kato Y, Lambert CA, Colige AC et al (2005) Acidic extracellular pH induces matrix metalloproteinase-9 expression in mouse metastatic melanoma cells through the phospholipase D-mitogen-activated protein kinase signaling. J Biol Chem 280:10938–10944. 10.1074/jbc.M411313200 15657063 10.1074/jbc.M411313200

[76] Khan ZS, Santos JM, Vaz NG, Hussain F (2019) Enhanced blebbing as a marker for metastatic prostate cancer. Biomicrofluidics 13:034110. 10.1063/1.5085346 31431812 10.1063/1.5085346 PMC6697032

[77] Paterson H, Reeves B, Brown R et al (1987) Activated N-ras controls the transformed phenotype of HT1080 human fibrosarcoma cells. Cell 51:803–812. 10.1016/0092-8674(87)90103-6 3315232 10.1016/0092-8674(87)90103-6

[78] Rayment I, Holden HM, Whittaker M et al (1993) Structure of the actin-myosin complex and its implications for muscle contraction. Science 261:58–65. 10.1126/science.8316858 8316858 10.1126/science.8316858

[79] Rayment I, Rypniewski WR, Schmidt-Bäse K et al (1993) Three-dimensional structure of myosin subfragment-1: a molecular motor. Science 261:50–58. 10.1126/science.8316857 8316857 10.1126/science.8316857

[80] Winkelmann DA, Almeda S, Vibert P, Cohen C (1984) A new myosin fragment: visualization of the regulatory domain. Nature 307:758–760. 10.1038/307758a0 6422307 10.1038/307758a0

[81] Côté GP, Robinson EA, Appella E, Korn ED (1984) Amino acid sequence of a segment of the *Acanthamoeba* myosin II heavy chain containing all three regulatory phosphorylation sites. J Biol Chem 259:12781–12787 6149217

[82] Angstadt S, Zhu Q, Jaffee EM et al (2022) Pancreatic ductal adenocarcinoma cortical mechanics and clinical implications. Front Oncol 12:809179. 10.3389/fonc.2022.809179 35174086 10.3389/fonc.2022.809179 PMC8843014

[83] Straight AF, Cheung A, Limouze J et al (2003) Dissecting temporal and spatial control of cytokinesis with a myosin II inhibitor. Science 299:1743–1747. 10.1126/science.1081412 12637748 10.1126/science.1081412

[84] Allingham JS, Smith R, Rayment I (2005) The structural basis of blebbistatin inhibition and specificity for myosin II. Nat Struct Mol Biol 12:378–379. 10.1038/nsmb908 15750603 10.1038/nsmb908

[85] Kovács M, Tóth J, Hetényi C et al (2004) Mechanism of blebbistatin inhibition of myosin II. J Biol Chem 279:35557–35563. 10.1074/jbc.M405319200 15205456 10.1074/jbc.M405319200

[86] Limouze J, Straight AF, Mitchison T, Sellers JR (2004) Specificity of blebbistatin, an inhibitor of myosin II. J Muscle Res Cell Motil 25:337–341. 10.1007/s10974-004-6060-7 15548862 10.1007/s10974-004-6060-7

[87] Cheung A, Dantzig JA, Hollingworth S et al (2002) A small-molecule inhibitor of skeletal muscle myosin II. Nat Cell Biol 4:83–88. 10.1038/ncb734 11744924 10.1038/ncb734

[88] Ramachandran I, Terry M, Ferrari MB (2003) Skeletal muscle myosin cross-bridge cycling is necessary for myofibrillogenesis. Cell Motil Cytoskeleton 55:61–72. 10.1002/cm.10113 12673599 10.1002/cm.10113

[89] Shaw MA, Ostap EM, Goldman YE (2003) Mechanism of inhibition of skeletal muscle actomyosin by N-benzyl-p-toluenesulfonamide. Biochemistry 42:6128–6135. 10.1021/bi026964f 12755615 10.1021/bi026964f

[90] Sellin LC, McArdle JJ (1994) Multiple effects of 2,3-butanedione monoxime. Pharmacol Toxicol 74:305–313. 10.1111/j.1600-0773.1994.tb01365.x 7937562 10.1111/j.1600-0773.1994.tb01365.x

[91] McKillop DF, Fortune NS, Ranatunga KW, Geeves MA (1994) The influence of 2,3-butanedione 2-monoxime (BDM) on the interaction between actin and myosin in solution and in skinned muscle fibres. J Muscle Res Cell Motil 15:309–318. 10.1007/BF00123483 7929796 10.1007/BF00123483

[92] Herrmann C, Wray J, Travers F, Barman T (1992) Effect of 2,3-butanedione monoxime on myosin and myofibrillar ATPases. An example of an uncompetitive inhibitor. Biochemistry 31:12227–12232. 10.1021/bi00163a036 1457420 10.1021/bi00163a036

[93] Wilson IB, Ginsburg B (1955) A powerful reactivator of alkylphosphate-inhibited acetylcholinesterase. Biochim Biophys Acta 18:168–170. 10.1016/0006-3002(55)90040-8 13260275 10.1016/0006-3002(55)90040-8

[94] Higuchi H, Takemori S (1989) Butanedione monoxime suppresses contraction and ATPase activity of rabbit skeletal muscle. J Biochem 105:638–643. 10.1093/oxfordjournals.jbchem.a122717 2527229 10.1093/oxfordjournals.jbchem.a122717

[95] Cramer LP, Mitchison TJ (1995) Myosin is involved in postmitotic cell spreading. J Cell Biol 131:179–189. 10.1083/jcb.131.1.179 7559774 10.1083/jcb.131.1.179 PMC2120601

[96] Fryer MW, Gage PW, Neering IR et al (1988) Paralysis of skeletal muscle by butanedione monoxime, a chemical phosphatase. Pflugers Arch 411:76–79. 10.1007/BF00581649 2832824 10.1007/BF00581649

[97] Stapleton MT, Fuchsbauer CM, Allshire AP (1998) BDM drives protein dephosphorylation and inhibits adenine nucleotide exchange in cardiomyocytes. Am J Physiol 275:H1260–1266. 10.1152/ajpheart.1998.275.4.H1260 9746474 10.1152/ajpheart.1998.275.4.H1260

[98] Somlyo AP, Somlyo AV (2003) Ca2 + sensitivity of smooth muscle and nonmuscle myosin II: modulated by G proteins, kinases, and myosin phosphatase. Physiol Rev 83:1325–1358. 10.1152/physrev.00023.2003 14506307 10.1152/physrev.00023.2003

[99] Chew TL, Masaracchia RA, Goeckeler ZM, Wysolmerski RB (1998) Phosphorylation of non-muscle myosin II regulatory light chain by p21-activated kinase (gamma-PAK). J Muscle Res Cell Motil 19:839–854. 10.1023/a:1005417926585 10047984 10.1023/a:1005417926585

[100] Zeng Q, Lagunoff D, Masaracchia R et al (2000) Endothelial cell retraction is induced by PAK2 monophosphorylation of myosin II. J Cell Sci 113(Pt 3):471–482. 10.1242/jcs.113.3.471 10639334 10.1242/jcs.113.3.471

[101] Heasman SJ, Ridley AJ (2008) Mammalian rho GTPases: new insights into their functions from in vivo studies. Nat Rev Mol Cell Biol 9:690–701. 10.1038/nrm2476 18719708 10.1038/nrm2476

[102] Vicente-Manzanares M, Ma X, Adelstein RS, Horwitz AR (2009) Non-muscle myosin II takes centre stage in cell adhesion and migration. Nat Rev Mol Cell Biol 10:778–790. 10.1038/nrm2786 19851336 10.1038/nrm2786 PMC2834236

[103] Georgouli M, Herraiz C, Crosas-Molist E et al (2019) Regional activation of myosin II in cancer cells drives tumor progression via a secretory cross-talk with the immune microenvironment. Cell 176:757-774e23. 10.1016/j.cell.2018.12.038 30712866 10.1016/j.cell.2018.12.038 PMC6370915

[104] Liu S, Goldstein RH, Scepansky EM, Rosenblatt M (2009) Inhibition of rho-associated kinase signaling prevents breast cancer metastasis to human bone. Cancer Res 69:8742–8751. 10.1158/0008-5472.CAN-09-1541 19887617 10.1158/0008-5472.CAN-09-1541

[105] Castoreno AB, Smurnyy Y, Torres AD et al (2010) Small molecules discovered in a pathway screen target the rho pathway in cytokinesis. Nat Chem Biol 6:457–463. 10.1038/nchembio.363 20436488 10.1038/nchembio.363 PMC2873065

[106] Patel RA, Liu Y, Wang B et al (2014) Identification of novel ROCK inhibitors with anti-migratory and anti-invasive activities. Oncogene 33:550–555. 10.1038/onc.2012.634 23396364 10.1038/onc.2012.634 PMC3977753

[107] Patel RA, Forinash KD, Pireddu R et al (2012) RKI-1447 is a potent inhibitor of the rho-associated ROCK kinases with anti-invasive and antitumor activities in breast cancer. Cancer Res 72:5025–5034. 10.1158/0008-5472.CAN-12-0954 22846914 10.1158/0008-5472.CAN-12-0954 PMC3463757

[108] Sun J, Zhang D, Zheng Y et al (2013) Targeting the metastasis suppressor, NDRG1, using novel iron chelators: regulation of stress fiber-mediated tumor cell migration via modulation of the ROCK1/pMLC2 signaling pathway. Mol Pharmacol 83:454–469. 10.1124/mol.112.083097 23188716 10.1124/mol.112.083097

[109] Liu X, Ji Q, Ye N et al (2015) Berberine inhibits invasion and metastasis of colorectal cancer cells via COX-2/PGE2 mediated JAK2/STAT3 signaling pathway. PLoS ONE 10:e0123478. 10.1371/journal.pone.0123478 25954974 10.1371/journal.pone.0123478 PMC4425560

[110] Barcelo J, Samain R, Sanz-Moreno V (2023) Preclinical to clinical utility of ROCK inhibitors in cancer. Trends Cancer 9:250–263. 10.1016/j.trecan.2022.12.001 36599733 10.1016/j.trecan.2022.12.001

[111] Kim S, Kim SA, Han J, Kim I-S (2021) Rho-kinase as a target for cancer therapy and its immunotherapeutic potential. Int J Mol Sci 22:12916. 10.3390/ijms222312916 34884721 10.3390/ijms222312916 PMC8657458

[112] Leloup L, Wells A (2011) Calpains as potential anti-cancer targets. Expert Opin Ther Targets 15:309–323. 10.1517/14728222.2011.553611 21244345 10.1517/14728222.2011.553611 PMC3076675

[113] Joshi VB, Gutierrez Ruiz OL, Razidlo GL (2023) The cell biology of metastatic invasion in pancreatic cancer: updates and mechanistic insights. Cancers (Basel) 15:2169. 10.3390/cancers15072169 37046830 10.3390/cancers15072169 PMC10093482

[114] Akgönüllü S, Bakhshpour M, Pişkin AK, Denizli A (2021) Microfluidic systems for cancer diagnosis and applications. Micromachines (Basel) 12:1349. 10.3390/mi12111349 34832761 10.3390/mi12111349 PMC8619454

[115] Och A, Och M, Nowak R et al (2022) Berberine, a herbal metabolite in the metabolic syndrome: the risk factors, course, and consequences of the disease. Molecules 27:1351. 10.3390/molecules27041351 35209140 10.3390/molecules27041351 PMC8874997

